# Ammonium dihydrogen (1-ammonio­pentane-1,1-di­yl)diphospho­nate

**DOI:** 10.1107/S1600536809028323

**Published:** 2009-07-22

**Authors:** Anatolij Dudko, Volodimir Bon, Alexandra Kozachkova, Vasily Pekhnyo

**Affiliations:** a Institute of General and Inorganic Chemistry, NAS Ukraine Kyiv, Prosp. Palladina 32/34, 03680 Ukraine

## Abstract

The title compound, NH_4_^+^·C_5_H_14_NO_6_P_2_^−^, was obtained from 1-ammonio-1-phosphono­pentane-1-phospho­nic acid and ammonium hydroxide in aqueous solution. The asymmetric unit of title compound contains one molecule, which consists of an ammonium cation and an aminodiphosphonic anion with the H atoms transferred from the phosphonic acid group to the amino group. The crystal structure shows a three-dimensional network of O—H⋯O and N—H⋯O hydrogen bonds which stabilize the structure.

## Related literature

For general background to the use of organic diphospho­nic acids as chelating agents in metal extraction and as drugs to prevent calcification and inhibit bone resorption, see: Matczak-Jon & Videnova-Adrabinska (2005 ▶); Tromelin *et al.* (1986 ▶); Szabo *et al.* (2002 ▶). For related structures, see: Bon *et al.* (2008 ▶). For bond–length data, see: Allen *et al.* (1987 ▶).
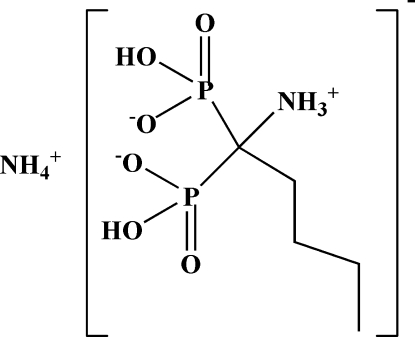


## Experimental

### 

#### Crystal data

NH_4_^+^·C_5_H_14_NO_6_P_2_^−^
*M* *_r_* = 264.15
Monoclinic, 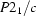

*a* = 9.6007 (6) Å
*b* = 5.7239 (4) Å
*c* = 20.3259 (15) Å
β = 98.100 (3)°
*V* = 1105.84 (13) Å^3^
*Z* = 4
Mo *K*α radiation
μ = 0.41 mm^−1^
*T* = 296 K
0.50 × 0.12 × 0.04 mm

#### Data collection

Bruker APEXII CCD diffractometer
Absorption correction: multi-scan (*SADABS*; Bruker, 2005 ▶) *T*_min_ = 0.824, *T*_max_ = 0.982
5297 measured reflections
2256 independent reflections
1532 reflections with *I* > 2σ(*I*)
*R*_int_ = 0.056

#### Refinement

*R*[*F* ^2^ > 2σ(*F* ^2^)] = 0.051
*wR*(*F* ^2^) = 0.122
*S* = 1.02
2256 reflections
173 parameters
H atoms treated by a mixture of independent and constrained refinement
Δρ_max_ = 0.37 e Å^−3^
Δρ_min_ = −0.38 e Å^−3^

### 

Data collection: *APEX2* (Bruker, 2005 ▶); cell refinement: *SAINT* (Bruker, 2005 ▶); data reduction: *SAINT*; program(s) used to solve structure: *SHELXS97* (Sheldrick, 2008 ▶); program(s) used to refine structure: *SHELXL97* (Sheldrick, 2008 ▶); molecular graphics: *SHELXTL* (Sheldrick, 2008 ▶); software used to prepare material for publication: *publCIF* (Westrip, 2009 ▶).

## Supplementary Material

Crystal structure: contains datablocks I, global. DOI: 10.1107/S1600536809028323/rk2155sup1.cif

Structure factors: contains datablocks I. DOI: 10.1107/S1600536809028323/rk2155Isup2.hkl

Additional supplementary materials:  crystallographic information; 3D view; checkCIF report

## Figures and Tables

**Table 1:** Hydrogen-bond geometry (Å, °)

*D*—H⋯*A*	*D*—H	H⋯*A*	*D*⋯*A*	*D*—H⋯*A*
N1—H11*N*⋯O2^i^	1.05 (4)	1.75 (4)	2.777 (4)	163 (3)
N1—H13*N*⋯O3^ii^	0.93 (4)	1.98 (4)	2.828 (4)	152 (3)
N1—H12*N*⋯O6^i^	0.93 (5)	2.08 (5)	2.879 (4)	143 (4)
O3—H3*O*⋯O5^ii^	0.80 (5)	1.72 (5)	2.519 (4)	174 (5)
O4—H4*O*⋯O6^iii^	0.82 (5)	1.75 (5)	2.566 (3)	173 (5)
N2—H22*N*⋯O1^iv^	0.86 (4)	1.95 (4)	2.781 (4)	161 (4)
N2—H21*N*⋯O2	1.02 (6)	1.77 (6)	2.769 (4)	165 (4)
N2—H23*N*⋯O5^v^	0.93 (6)	2.07 (6)	2.787 (5)	134 (5)
N2—H24*N*⋯O1^vi^	0.92 (5)	1.83 (5)	2.705 (5)	159 (4)

Symmetry codes: (i) 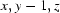; (ii) 
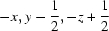
; (iii) 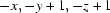; (iv) 
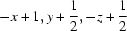
; (v) 
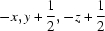
; (vi) 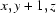.

**Fig. 1.**
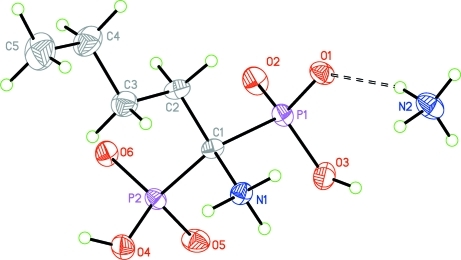
The asymmetric unit of title compound with the atom numbering scheme. Displacement ellipsoids are drawn at 50% probability level. Hydrogen atoms are presented as a small spheres of arbitrary rAdius.

**Fig. 2.**
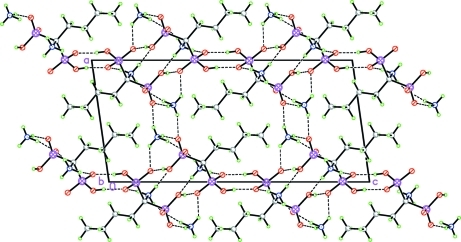
Crystal packing of title compound, projection along b axis. Dashed lines indicate hydrogen bonds.

## supplementary crystallographic information

### Comment

The organic diphosphonic acids are potentially very powerful chelating agents used in metal extractions and are tested by the pharmaceutical industry for use as efficient drugs preventing calcification and inhibiting bone resorption (Tromelin *et al.*, 1986, Matczak-Jon & Videnova-Adrabinska, 2005). Diphosphonic acids are used in the treatment of Paget disease, osteoporosis and tumoral osteolysis (Szabo *et al.*, 2002). The asymmetric unit of title compound (Fig. 1) contains one molecule, which exists as anion with protons transferred from the phosphonic group to the amino group. The ammonium cation attendant in structure neutralizes the negatively charged phosphonic acid residual. The phosphorus atom displays a slightly distorted tetrahedral geometry provided by three oxygen atoms and one carbon atom. Bond lengths and angles have normal values (Allen *et al.*, 1987). The crystal structure of title compound shows three–dimensional network of O—H···O and N—H···O hydrogen bonds which additionally stabilized the structure (Table 1, Fig. 2).

### Experimental

The title compound was obtained by the reaction of 1–ammonio–1–phosphonopentane–1–phosphonic acid and ammonium hydroxide (1:1) in the aqueous solution. The solution was left at room temperature. Colourless crystals of the title compound were obtained after 5 days staying.

### Refinement

The H atoms bonded to O and N atoms were located in a difference map and refined freely. Other H atoms which bonded to C were positioned geometrically and refined using a riding model with C—H = 0.96 Å for CH_3_ with *U*_iso_(H) = 1.5*U*_eq_(C) and C—H = 0.97 Å for CH_2_ with *U*_iso_(H) = 1.2*U*_eq_(C).

### Crystal data

**Table:**

NH_4_^+^·C_5_H_14_NO_6_P_2_^−^	*F*(000) = 560
*M**_r_* = 264.15	*D*_x_ = 1.587 Mg m^−^^3^
Monoclinic, *P*2_1_/*c*	Melting point: 495 K
Hall symbol: -P 2ybc	Mo *K*α radiation, λ = 0.71073 Å
*a* = 9.6007 (6) Å	Cell parameters from 824 reflections
*b* = 5.7239 (4) Å	θ = 2.7–21.1°
*c* = 20.3259 (15) Å	µ = 0.41 mm^−^^1^
β = 98.100 (3)°	*T* = 296 K
*V* = 1105.84 (13) Å^3^	Needle, colourless
*Z* = 4	0.50 × 0.12 × 0.04 mm

### Data collection

**Table:**

Bruker APEXII CCD diffractometer	2256 independent reflections
Radiation source: fine–focus sealed tube	1532 reflections with *I* > 2σ(*I*)
graphite	*R*_int_ = 0.056
Detector resolution: 8.26 pixels mm^-1^	θ_max_ = 26.4°, θ_min_ = 2.0°
φ and ω scans	*h* = −11→11
Absorption correction: multi-scan (*SADABS*; Bruker, 2005)	*k* = −7→5
*T*_min_ = 0.824, *T*_max_ = 0.982	*l* = −24→25
5297 measured reflections	

### Refinement

**Table:**

Refinement on *F*^2^	Primary atom site location: structure-invariant direct methods
Least-squares matrix: full	Secondary atom site location: difference Fourier map
*R*[*F*^2^ > 2σ(*F*^2^)] = 0.051	Hydrogen site location: inferred from neighbouring sites
*wR*(*F*^2^) = 0.122	H atoms treated by a mixture of independent and constrained refinement
*S* = 1.02	*w* = 1/[σ^2^(*F*_o_^2^) + (0.0435*P*)^2^ + 0.4298*P*] where *P* = (*F*_o_^2^ + 2*F*_c_^2^)/3
2256 reflections	(Δ/σ)_max_ < 0.001
173 parameters	Δρ_max_ = 0.37 e Å^−^^3^
0 restraints	Δρ_min_ = −0.38 e Å^−^^3^

### Special details

**Table:**

Geometry. All s.u.'s (except the s.u. in the dihedral angle between two l.s. planes) are estimated using the full covariance matrix. The cell s.u.'s are taken into account individually in the estimation of s.u.'s in distances, angles and torsion angles; correlations between s.u.'s in cell parameters are only used when they are defined by crystal symmetry. An approximate (isotropic) treatment of cell s.u.'s is used for estimating s.u.'s involving l.s. planes.
Refinement. Refinement of *F*^2^ against ALL reflections. The weighted *R*–factor *wR* and goodness of fit *S* are based on *F*^2^, conventional *R*–factors *R* are based on *F*, with *F* set to zero for negative *F*^2^. The threshold expression of *F*^2^ > 2σ(*F*^2^) is used only for calculating *R*–factors(gt) *etc*. and is not relevant to the choice of reflections for refinement. *R*–factors based on *F*^2^ are statistically about twice as large as those based on *F*, and *R*–factors based on ALL data will be even larger.

### Fractional atomic coordinates and isotropic or equivalent isotropic displacement parameters (Å^2^)

**Table:**

	*x*	*y*	*z*	*U*_iso_*/*U*_eq_	
P1	0.22481 (10)	0.43513 (17)	0.29915 (4)	0.0195 (3)	
P2	0.00587 (9)	0.47871 (16)	0.39608 (4)	0.0184 (2)	
C1	0.1590 (3)	0.3149 (6)	0.37310 (16)	0.0161 (7)	
C2	0.2832 (4)	0.3109 (7)	0.43011 (17)	0.0243 (8)	
H2A	0.3635	0.2439	0.4129	0.029*	
H2B	0.3070	0.4715	0.4421	0.029*	
C3	0.2649 (4)	0.1806 (7)	0.49344 (17)	0.0295 (9)	
H3A	0.1787	0.2316	0.5088	0.035*	
H3B	0.2567	0.0146	0.4841	0.035*	
C4	0.3877 (4)	0.2227 (8)	0.54735 (18)	0.0374 (11)	
H4A	0.3885	0.3860	0.5602	0.045*	
H4B	0.4745	0.1909	0.5297	0.045*	
C5	0.3827 (5)	0.0731 (9)	0.6084 (2)	0.0506 (13)	
H5A	0.2985	0.1074	0.6271	0.076*	
H5B	0.4634	0.1059	0.6407	0.076*	
H5C	0.3831	−0.0889	0.5962	0.076*	
N1	0.1106 (3)	0.0689 (5)	0.35619 (16)	0.0200 (7)	
N2	0.3682 (4)	0.9144 (7)	0.2109 (2)	0.0297 (8)	
O1	0.3514 (2)	0.2924 (4)	0.28929 (11)	0.0247 (6)	
O2	0.2493 (3)	0.6898 (4)	0.31012 (12)	0.0279 (6)	
O3	0.1056 (3)	0.3928 (5)	0.23993 (12)	0.0282 (7)	
O4	−0.0725 (3)	0.2932 (4)	0.43385 (13)	0.0263 (6)	
O5	−0.0902 (2)	0.5374 (5)	0.33383 (12)	0.0280 (6)	
O6	0.0601 (3)	0.6800 (4)	0.43945 (11)	0.0237 (6)	
H3O	0.107 (5)	0.279 (9)	0.217 (2)	0.065 (18)*	
H4O	−0.061 (5)	0.303 (9)	0.475 (2)	0.070 (18)*	
H11N	0.180 (4)	−0.059 (7)	0.3428 (19)	0.047 (12)*	
H12N	0.078 (5)	−0.001 (8)	0.392 (2)	0.060 (14)*	
H13N	0.038 (4)	0.063 (7)	0.321 (2)	0.040 (12)*	
H21N	0.336 (5)	0.810 (9)	0.247 (3)	0.083 (18)*	
H22N	0.449 (5)	0.874 (7)	0.2012 (19)	0.040 (13)*	
H23N	0.295 (6)	0.919 (10)	0.176 (3)	0.10 (2)*	
H24N	0.377 (5)	1.058 (8)	0.231 (2)	0.048 (14)*	

### Atomic displacement parameters (Å^2^)

**Table:**

	*U*^11^	*U*^22^	*U*^33^	*U*^12^	*U*^13^	*U*^23^
P1	0.0216 (5)	0.0158 (5)	0.0220 (5)	−0.0005 (4)	0.0066 (4)	0.0005 (4)
P2	0.0181 (5)	0.0165 (5)	0.0213 (5)	0.0008 (4)	0.0054 (4)	0.0007 (4)
C1	0.0180 (17)	0.0085 (18)	0.0212 (17)	−0.0008 (15)	0.0006 (14)	0.0005 (13)
C2	0.0189 (18)	0.026 (2)	0.0263 (19)	−0.0043 (17)	−0.0025 (15)	−0.0006 (16)
C3	0.028 (2)	0.034 (3)	0.0250 (19)	−0.002 (2)	0.0005 (17)	0.0037 (17)
C4	0.037 (2)	0.043 (3)	0.029 (2)	0.004 (2)	−0.0054 (19)	0.0001 (19)
C5	0.052 (3)	0.068 (4)	0.030 (2)	0.011 (3)	0.000 (2)	0.007 (2)
N1	0.0237 (16)	0.0152 (17)	0.0211 (16)	−0.0026 (14)	0.0028 (14)	−0.0007 (13)
N2	0.026 (2)	0.026 (2)	0.040 (2)	0.0035 (18)	0.0115 (18)	0.0002 (17)
O1	0.0194 (13)	0.0244 (15)	0.0314 (13)	0.0026 (12)	0.0074 (11)	−0.0023 (11)
O2	0.0360 (15)	0.0154 (15)	0.0347 (14)	−0.0015 (13)	0.0132 (12)	−0.0007 (11)
O3	0.0297 (15)	0.0329 (18)	0.0216 (13)	0.0049 (14)	0.0019 (12)	−0.0045 (13)
O4	0.0315 (15)	0.0229 (16)	0.0265 (15)	−0.0089 (12)	0.0108 (12)	−0.0013 (12)
O5	0.0198 (12)	0.0329 (17)	0.0305 (14)	0.0030 (12)	0.0002 (11)	0.0063 (12)
O6	0.0317 (14)	0.0131 (14)	0.0278 (13)	−0.0043 (12)	0.0097 (11)	−0.0040 (10)

### Geometric parameters (Å, °)

**Table:**

P1—O2	1.488 (3)	C4—C5	1.514 (5)
P1—O1	1.501 (2)	C4—H4A	0.9700
P1—O3	1.559 (3)	C4—H4B	0.9700
P1—C1	1.844 (3)	C5—H5A	0.9600
P2—O5	1.495 (2)	C5—H5B	0.9600
P2—O6	1.499 (2)	C5—H5C	0.9600
P2—O4	1.564 (3)	N1—H11N	1.05 (4)
P2—C1	1.858 (3)	N1—H12N	0.93 (5)
C1—N1	1.507 (4)	N1—H13N	0.93 (4)
C1—C2	1.541 (5)	N2—H21N	1.02 (6)
C2—C3	1.519 (5)	N2—H22N	0.86 (4)
C2—H2A	0.9700	N2—H23N	0.93 (6)
C2—H2B	0.9700	N2—H24N	0.92 (5)
C3—C4	1.511 (5)	O3—H3O	0.80 (5)
C3—H3A	0.9700	O4—H4O	0.82 (5)
C3—H3B	0.9700		
			
O2—P1—O1	116.04 (14)	C2—C3—H3B	109.4
O2—P1—O3	110.56 (16)	H3A—C3—H3B	108.0
O1—P1—O3	109.44 (15)	C3—C4—C5	113.1 (4)
O2—P1—C1	107.97 (15)	C3—C4—H4A	109.0
O1—P1—C1	106.42 (15)	C5—C4—H4A	109.0
O3—P1—C1	105.83 (15)	C3—C4—H4B	109.0
O5—P2—O6	116.53 (15)	C5—C4—H4B	109.0
O5—P2—O4	106.61 (15)	H4A—C4—H4B	107.8
O6—P2—O4	112.60 (14)	C4—C5—H5A	109.5
O5—P2—C1	108.42 (14)	C4—C5—H5B	109.5
O6—P2—C1	108.33 (14)	H5A—C5—H5B	109.5
O4—P2—C1	103.51 (14)	C4—C5—H5C	109.5
N1—C1—C2	109.8 (3)	H5A—C5—H5C	109.5
N1—C1—P1	107.0 (2)	H5B—C5—H5C	109.5
C2—C1—P1	107.5 (2)	C1—N1—H11N	122 (2)
N1—C1—P2	107.4 (2)	C1—N1—H12N	110 (3)
C2—C1—P2	112.0 (2)	H11N—N1—H12N	101 (3)
P1—C1—P2	113.01 (17)	C1—N1—H13N	113 (2)
C3—C2—C1	118.3 (3)	H11N—N1—H13N	102 (3)
C3—C2—H2A	107.7	H12N—N1—H13N	107 (4)
C1—C2—H2A	107.7	H21N—N2—H22N	112 (4)
C3—C2—H2B	107.7	H21N—N2—H23N	107 (4)
C1—C2—H2B	107.7	H22N—N2—H23N	116 (4)
H2A—C2—H2B	107.1	H21N—N2—H24N	103 (4)
C4—C3—C2	111.4 (3)	H22N—N2—H24N	108 (4)
C4—C3—H3A	109.4	H23N—N2—H24N	109 (4)
C2—C3—H3A	109.4	P1—O3—H3O	120 (4)
C4—C3—H3B	109.4	P2—O4—H4O	116 (4)
			
O2—P1—C1—N1	170.1 (2)	O5—P2—C1—C2	162.9 (2)
O1—P1—C1—N1	−64.7 (2)	O6—P2—C1—C2	35.6 (3)
O3—P1—C1—N1	51.7 (3)	O4—P2—C1—C2	−84.2 (3)
O2—P1—C1—C2	−72.0 (3)	O5—P2—C1—P1	41.3 (2)
O1—P1—C1—C2	53.2 (3)	O6—P2—C1—P1	−86.01 (19)
O3—P1—C1—C2	169.6 (2)	O4—P2—C1—P1	154.25 (17)
O2—P1—C1—P2	52.1 (2)	N1—C1—C2—C3	−53.3 (4)
O1—P1—C1—P2	177.27 (16)	P1—C1—C2—C3	−169.4 (3)
O3—P1—C1—P2	−66.4 (2)	P2—C1—C2—C3	65.9 (4)
O5—P2—C1—N1	−76.5 (2)	C1—C2—C3—C4	−171.9 (3)
O6—P2—C1—N1	156.2 (2)	C2—C3—C4—C5	−172.9 (3)
O4—P2—C1—N1	36.4 (2)		

### Hydrogen-bond geometry (Å, °)

**Table:**

*D*—H···*A*	*D*—H	H···*A*	*D*···*A*	*D*—H···*A*
N1—H11N···O2^i^	1.05 (4)	1.75 (4)	2.777 (4)	163 (3)
N1—H13N···O3^ii^	0.93 (4)	1.98 (4)	2.828 (4)	152 (3)
N1—H12N···O6^i^	0.93 (5)	2.08 (5)	2.879 (4)	143 (4)
O3—H3O···O5^ii^	0.80 (5)	1.72 (5)	2.519 (4)	174 (5)
O4—H4O···O6^iii^	0.82 (5)	1.75 (5)	2.566 (3)	173 (5)
N2—H22N···O1^iv^	0.86 (4)	1.95 (4)	2.781 (4)	161 (4)
N2—H21N···O2	1.02 (6)	1.77 (6)	2.769 (4)	165 (4)
N2—H23N···O5^v^	0.93 (6)	2.07 (6)	2.787 (5)	134 (5)
N2—H24N···O1^vi^	0.92 (5)	1.83 (5)	2.705 (5)	159 (4)

Symmetry codes: (i) *x*, *y*−1, *z*; (ii) −*x*, *y*−1/2, −*z*+1/2; (iii) −*x*, −*y*+1, −*z*+1; (iv) −*x*+1, *y*+1/2, −*z*+1/2; (v) −*x*, *y*+1/2, −*z*+1/2; (vi) *x*, *y*+1, *z*.
